# Investigating the beliefs of Saudi females regarding physical activity: a qualitative exploration

**DOI:** 10.1080/17482631.2023.2296696

**Published:** 2023-12-21

**Authors:** Basmah Fehaid H Alharbi, Philip Baker, Toby Pavey, Manal F. Alharbi

**Affiliations:** a Basic Health Science Department, Qassim University, Applied Medical Science College, Al-Qassim Province, Kingdom of Saudi Arabia; b Faculty of Health, School of Public Health and Social Work, Queensland University of Technology, Kelvin Grove; c Faculty of Health, School of Exercise and Nutrition Sciences, Queensland University of Technology; d Maternal & Child Health Nursing Department, College of Nursing, King Saud University, Riyadh, Saudi Arabia

**Keywords:** Physical activity, Saudi Arabia, females, qualitative research, Psychosocial factors, Environmental factors

## Abstract

**Purpose:**

Saudi women have been deprived of equal access to and opportunities for an active lifestyle due to sociocultural restrictions. Using the theory of planned behaviour, this elicitation study aimed to explore the salient beliefs of young Saudi women aged 18–35 regarding physical activity (PA).

**Methods:**

A descriptive qualitative methodology was utilized using a semi-structured interview. A total of 25 transcribed interviews were coded. Content analysis was used to identify the salient beliefs and to rank-order the beliefs using an adaptation of Burnard’s model.

**Findings:**

Thirty-five salient beliefs were identified as dominant factors in the decision to engage in PA. The salient beliefs about PA are classified as positive (related to advantages, social approval, and enabling) and negative (related to disadvantages, social disapproval, and barriers). These included behavioural beliefs (concerning physical/psychological well-being and social opportunities), normative beliefs (concerning family, community, international and local media influencers’ expectations), and control beliefs (concerning personal, social, natural, and built environment enablers as well as constraints).

**Conclusions:**

Due to the elicited beliefs, young women may be able to adopt more active lifestyles and change their inactive behaviour. Addressing negative beliefs can help change their inactive behaviour. Strengthening positive beliefs and facilitators is also beneficial.

## Background

Living an active lifestyle is associated with many health and well-being outcomes, including decreased risk of cardiovascular disease, type 2 diabetes, obesity, cancer, depression, and mortality and improved overall quality of life (Bull & Bauman, 2011; Warburton et al., 2006). However, insufficient adult physical activity (PA) levels are increasing, with the 2016 age-standardized prevalence of global PA at 27.5%, not meeting the WHO global PA guidelines (Guthold et al., 2018). These levels of physical inactivity remain a public health problem (Trost et al., 2014) and contribute significantly to both global disease and economic burden (Andersen et al., 2016; Ding et al., 2016). In the Kingdom of Saudi Arabia (KSA), the prevalence of physical inactivity was 53% (Guthold et al., 2018), with an economic burden of US $1.40 billion on the healthcare system in 2013 (Ding et al., 2016). Furthermore, a gender divide is evident in the Saudi population as the prevalence of physical inactivity is 20% less for males than females (45% vs. 65%) (Guthold et al., 2018). A cross-sectional study identified that a lack of designated areas for PA and encouragement hinder PA among female university students (Samara et al., 2015). A systematic analysis of cross-sectional research (Al-Hazzaa, 2018) found around 75% of Saudi women aged 18 years and older are physically inactive. A recent mixed-method study that assessed PA among female university students revealed that 70% of participants did not meet the WHO recommendation of 150 minutes per week of moderate activity, while around 62% of participants did not meet the WHO recommendation of 75 minutes per week of vigorous activity (Aljehani et al., 2022).

Additionally, the study identified inadequate PA facilities, academic workload, gender roles, and the need to uphold cultural standards as barriers. Facilitators were valuing positive outcomes, general health concerns, and family support for PA involvement (Aljehani et al., 2022). Accordingly, a large number of young Saudi women are at a higher risk for developing noncommunicable diseases according to the age distribution in this population (70% are aged 18 to 30 years) (Alahmed & Lobelo, 2017; Alshaikh et al., 2016).

Given the high prevalence of physical inactivity among young Saudi women, there is an urgent need to prioritize enhancing PA policies and programmes. Within the national public policy context, Vision 2030 embraced an empowerment approach, including removing barriers to women with an initiative to support sports as well as physical activities and increase the exercise rate of Saudis by 40% (exercising once a week) (Vision). Vision 2030 is a national reform strategy for decreasing Saudi Arabia’s dependency on oil, diversifying the Saudi economy, and enhancing public service sectors including health, education, infrastructure, leisure, and tourism (Vision).

Increasing PA among this population is difficult due to long-term restrictions and roles enforced through legal and societal measures. Culturally, Saudi women are not encouraged to perform or engage in any outdoor exercise except walking (Aljehani et al., 2022; Sharara et al., 2018). Changing beliefs and opinions, then practices, may in turn improve health.

Numerous studies have shown several sociodemographic and socioeconomic characteristics associated with PA among Saudi females. The factors including advancing age (Al-Hazzaa, 2018); gender (female) (Al-Hazzaa, 2018; Amin et al., 2011, 2012; Al-Nozha et al., 2007); the presence of chronic disease (Amin et al., 2012); residence setting (urban) (Al-Hazzaa, 2018; Amin et al., 2012); education levels; and occupational status (Al-Hazzaa, 2018; Amin et al., 2011) are believed to be negatively associated with Saudi female PA. Conversely, marital status and parents’ educational levels (Khalaf et al., 2013) are thought to be positively related to Saudi female PA. In the literature, many have examined psychosocial factors including knowledge of PA benefits, attitude, past behaviour, pros and cons, and self-efficacy, which, in turn, may have an impact on PA (Mabry et al., 2016; Prince et al., 2014). However, if promoters fail to study the beliefs regarding harmful effects and enablers of PA, the likelihood of successfully encouraging physically active behaviour is minimal (Lox et al., 2017; Tomasone et al., 2014). Amin et al. (2011) found that traditions (e.g., lack of family support, being a female who is supposed to prioritize their family, and social restrictions) were reported as the leading barrier (80% of responses) to leisure time PA (Amin et al., 2011). Another conclusion was that a lack of social support and support from friends was a deterrent to being active (Al-Otaibi, 2013; AlQuaiz & Tayel, 2009; Awadalla et al., 2014). However, there is a lack of evidence regarding other social factors, such as different groups young Saudi women encounter, for example, mothers, siblings, or cousins, who may support engagement in an active lifestyle. Therefore, health advocates are less equipped to effectively create evidence-based interventions that could raise Saudi women’s PA levels.

Several studies cited environmental level factors influence PA among Saudi females aged 18 years and over (AlQuaiz & Tayel, 2009; Alsahli, 2016; Amin et al., 2011; Awadalla et al., 2014; Khalaf et al., 2013; Samara et al., 2015). Publications that focused on researching environmental elements that correlate with PA behaviour reported that the “lack of accessible sports facilities” and “weather” were the two most prevalent impediments. It is noted that the most likely PA barrier for young Saudi females is a lack of facilities (80.5%), a score significantly higher in females compared to their male counterparts (AlQuaiz & Tayel, 2009). Likewise, these results are consistent with the results of Samara et al. (2015), which showed lack of suitable spaces for engaging in PA and exercise as the biggest obstacle to PA among the target group (Samara et al., 2015).

Social-cognitive influences and environmental factors are more vital determinants of PA engagement than biological causes (28 (Alahmed & Lobelo, 2018). Saudi women’s perceptions of personal and socio-environmental circumstances can be altered by releasing them from cultural limitations and PA-inhibiting variables such as lack of family support, being a female who is expected to prioritize her family, and social restrictions (Samara et al., 2015; Sharara et al., 2018). Social and political change may offer opportunities to implement new PA strategies that can close the physical inactivity gender gap, improve health, and reduce the burden of disease as well as associated costs related to physical inactivity.

A systematic review of PA and sedentary lifestyle to identify the need for prevention and policy-related research conducted in the cooperating oil-producing countries (the KSA, Bahrain, Kuwait, Oman, Qatar, and the United Arab Emirates) found a dearth of studies describing the psychosocial factors associated with active behaviour in a theoretical behaviour change framework (Dishman, 2008). Theoretical frameworks can explain an individual’s perceptions; however, the theoretical points of reference must be clarified. An application of a health behaviour theoretical framework could better understand factors that influence PA behaviour for young Saudi women and subsequently lead to appropriate interventions to promote PA levels. The theory of planned behaviour (TPB), a theory frequently used to forecast an individual’s propensity to adopt a specific activity (Dishman et al., 2013), provides such reference points.

The TPB has the potential to reveal the factors influencing Saudi women’s PA behaviour. It specifies the dimensions of attitude, subjective norm, and perceived behavioural control as reliable predictors of PA intention and PA behaviour (32 (Al-Harbi & Al-Harbi, 2017). Attitude refers to the positive or negative values the individual holds towards the performance of the behaviour. Subjective norms refer to the perceived social pressure to engage or not in behaviour. Perceived behavioural control represents the individual’s perceptions of the presence or absence of enablers and barriers to performing the behaviour. Those conceptions are generated in an individual by salient beliefs regarding a particular behaviour. Thus, the whole TPB is based on salient beliefs. Downs and Hausenblas (2005) published a review of elicitation experiments in the PA domain (Downs & Hausenblas, 2005). An assessment of 47 TPB publications that identified the most salient behavioural, normative, and control beliefs related to PA participation was conducted. It is evident from the study findings that elicitation investigations are crucial for comprehending PA behaviour. TPB argues that salient belief elicitation is critical to understanding the behaviour of a target group (Mabry et al., 2016).

Little is known about young Saudi women’s intentions and planned behaviour towards PA. To examine PA intentions and behaviour, it is crucial to elicit young Saudi women’s beliefs about PA behaviour and the environment. Elicitation studies are advised when applying planned behaviour (Al-Harbi & Al-Harbi, 2017). In Saudi Arabia, elicitation studies are rare, and only two have identified the salient beliefs underpinning PA behaviour following global guidelines in young women (Al-Harbi & Al-Harbi, 2017; Saaty et al., 2015). Al-Harbi and Al-Harbi identified 21 salient beliefs from a sample of 25 young Saudi women (aged 18–20) and included behavioural beliefs (e.g., PA relieves stress), normative beliefs (derived, e.g., from social media), and control beliefs (e.g., weather and lack of suitable female facilities are obstacles to PA) (Al-Harbi & Al-Harbi, 2017; Saaty et al., 2015). To support the aims of Vision 2030, knowledge of women’s salient beliefs about PA is a priority. Thus, using TPB as a framework, this study investigates young Saudi women’s salient beliefs regarding PA participation. In this study, we aim to explore the importance of the salient beliefs of young Saudi women when it comes to participating in physical activities. By probing into their perspectives, we hope to better understand their views and attitudes towards engaging in physical activity. By shedding light on these key beliefs, valuable insights can be gained to inform strategies and interventions to promote and encourage PA among young Saudi women.

## Methods

### Study design and participants

A descriptive qualitative approach was utilized using a semi-structured interview of open-ended questions with individual participants who gave written informed consent. The sample was recruited between October and December 2018 from a public university in Saudi Arabia. Twenty-five young Saudi females (18–35 years old) were recruited from King Saud University (KSU) following elicitation study size suggestions (*N* ≥ 25) (Francis et al., 2004). Interviews elicited the TPB’s three constructs on salient behavioural, normative, and control beliefs.

### Data collection

Participants were recruited using a convenience sampling method through email invitations extended by KSU. The Scientific Research Department emailed the entire potentially eligible target population 24,136 Saudi women regardless of age (University, 2020), with an invitation letter. As only 25 participants were needed, the participants were advised that only the first 25 responders would be interviewed and that the researcher would cease taking consent replies once the positions had been filled. A Saudi female from Australia conducted Skype interviews for time, cost, and geographical efficiency Fields (Janghorban et al., 2014). Consenting participants were sent a Skype contact request for a scheduled interview—the semi-structured interview in Arabic comprised nine open-ended questions as presented in Table I.

**Table I.** Interview questions.

Behavioural beliefs questions	
Question 1	What do you believe are the pros of doing physical activity?
Question 2	What do you believe are the cons of doing physical activity?
Question 3	What else comes to mind when you think about doing physical activity?
Normative beliefs questions:	
Question 1	Which people would approve or support you doing physical activity?
Question 2	Which people would disapprove of you doing physical activity?
Question 3	Are there any other groups/people who come to mind when you think about engaging in physical activity?
Control beliefs questions	
Question 1	What circumstances would enable you to be physically active?
Question 2	What circumstances would deter you from performing physical activity?
Question 3	Are there any other issues that come to mind when you think about the difficulty of engaging in physical activity?

Each conversation began with a scenario that explained PA related objectives of Saudi’s Vision 2030, as shown in Supplementary 1. Questions were constructed to examine the salient beliefs of young Saudi women regarding PA. The PA behaviour definition was based on global recommendations of the World Health Organization (World Health Organisation, 2018) for healthy adults aged 18 to 64 years: that is, engaging in 150 minutes of moderate-intensity aerobic PA throughout the week or engaging in at least 75 minutes of vigorous-intensity aerobic PA throughout the week or a combination of both forms. Interview protocol probes were used where necessary to encourage interviewees to explain or expand their opinions (Supplementary 1). The interview guide and open-ended questions were piloted with one interviewee to gauge the clarity of the questions, the effectiveness of Skype in an interview setting, and the amount of time needed to interview the participants. Also, the interview guide and open-ended questions have been tested with 24 interviewees. This step was tested, piloted, and published in a prior elicitation study by one of the authors (Al-Harbi & Al-Harbi, 2017).

Each recorded interview, 30- to 45-minute duration, was transcribed verbatim. Shortly after the interview, the interviewees checked the transcripts for correctness, and subsequently, the subject’s name was changed to a unique identifier.

### Data analysis

Participants’ responses were themed and coded and then categorized as either positive or negative salient beliefs. Ajzen (2006) recommends using content analysis to determine the participant’s salient beliefs (Ajzen, 2006b). Content analysis can be conducted using either an inductive or a deductive approach (Elo et al., 2014). In this research, both deductive and inductive methods were applied. Utilizing the TPB beliefs-guided script (which refers to behavioural beliefs, normative beliefs, and control belief constructs) reflects a deductive approach. Constructing themes and subthemes that arose from the data reflects an inductive technique. Content analysis identified the salient beliefs (Elo et al., 2014) using an adaptation of Burnard’s model (Burnard, 1991) to construct themes subjective to a) advantages and disadvantages’ outcomes, b) lists of people who approve and disapprove of PA, and c) salient circumstances that enable and hinder PA.

An independent exercise psychologist evaluated the validation and clarification of themes and directed the thematic outcomes. The salient beliefs were categorized by gathering responses of a similar outcome and noting the response frequency by at least 20% of the participant’s (Icek Ajzen, 1980). Each salient belief’s orientation is classified as either positive (when categorized under the advantages outcomes, social referents approving of PA, enablers to PA) or negative (when categorized under the disadvantages outcomes, the social referents disapproving of PA, barriers to PA). Overall, the transcripts were analysed within the TPB framework.

### Translation process

**Figure 1.**
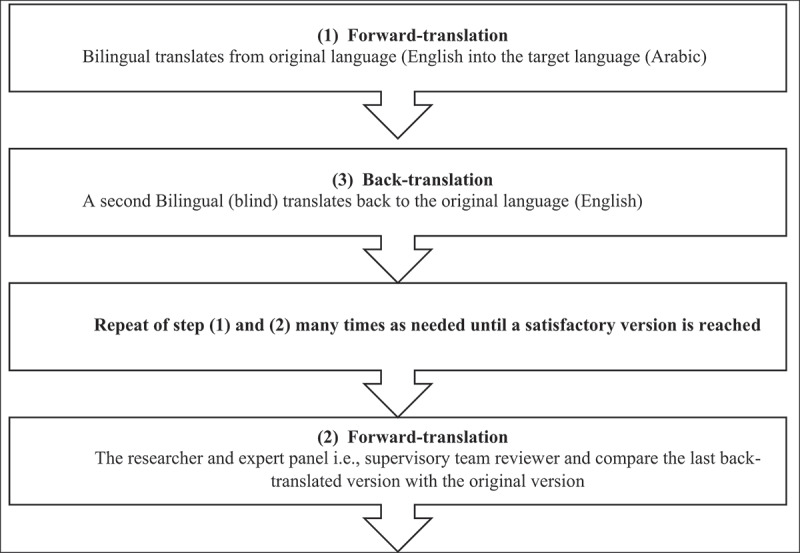
Brislin’s translation steps (Brislin, 1986).

Interview materials were forward- and backward-translated by two English—Arabic specialists, assisted by an adaptation of Brislin’s translation model (Brislin, 1986) to reduce cross-cultural prejudice (Regmi et al., 2010) and to ensure interchangeability of the translated materials (Cha et al., 2007). The researcher and the panel subsequently compared the two versions and then finalized when no further modifications were required. An adequate translation process is shown in Figure 1.

The participants’ transcripts were analysed in the participants’ and the researcher’s first language, Arabic, to validate the findings, as recommended by Nikander (Nikander, 2008). The analysis was aided by an adaptation of the framework of Chen and Boore (Chen & Boore, 2010). This framework involved analysing only the data in the original language and translating the analysed portion (data) to the target language (English) according to the usual standards of the forward and backward translation process. The themes and subthemes were forward-translated, from Arabic to English, by a panel committee including the researcher and a certified specialist of both English and Arabic languages as well as Saudi culture. The themes and subthemes were backward-translated, English to Arabic, by an independent bilingual Saudi, proficient in both languages. After comparing the two versions, enhancements for clarity and simplicity were incorporated into the completed findings.

### Ethical statement

The Ethics Committee of the Queensland University of Technology and the Scientific Research Department of King Saud University have given their approval for this study. All procedures conducted in the study that involved human participants followed the ethical standards set by institutional and national research committees, as well as the 1964 Helsinki Declaration and its subsequent amendments or equivalent ethical standards. Before the interviews, all participants provided their informed consent.

## Results

### Sample characteristics

Over a 3-month recruitment and interview period, 25 Saudi female participants aged 18–35 participated in the study. Most respondents were young women between the ages of 18 and 24 (80%) and single without children (96%). Regarding their current level of education, 80% were undergraduates, while the remainder were postgraduates, as presented in Table II.

**Table II.** Demographic characteristics of the interviewees (*N* = 25).

Characteristics	Cateogry	Frequencies	%
Age (Years)	18–24 years old	20	80%
	25–35 years old	5	20%
Marital Status	Single	24	96%
	Married	1	4%
Education Level	Undergraduate	20	80%
	Postgraduate	5	20%

### Behavioral beliefs

Table III shows the behavioural belief themes, salient beliefs, and the frequency of these beliefs reported by the participants. Overall, 15 salient behavioural beliefs were categorized under 6 main themes. Enhancing physical well-being was the highest endorsed theme. Among the six key health-related beliefs identified, improving fitness level was the most frequently endorsed belief. Having an ideal weight, enhancing one’s self-perception, improving general health, and reducing chronic disease risk were all strongly recommended, respectively. Comments related to physical well-being include the following:

**Table III.** Salient behavioural beliefs among young Saudi females aged 18–35 years regarding physical activity based on global guidelines.

Themes	Rank	Sub-themes	Frequency %
**The advantages of doing the PA**			
Physical wellbeing	1	Increasing fitness level	17 (68%)
	2	Having an ideal body weight	16 (64%)
	3	Improving body image	15 (60%)
	4	Reducing the risk of chronic diseases	15 (60%)
	5	Improving health	14 (56%)
	6	Strengthening muscles and body	6 (24%)
Psychological wellbeing	1	Reducing current stress and anxiety	12 (48%)
	2	Increasing self-confidence	11 (44%)
	3	Relieving negative feelings	6 (24%)
	4	Improving mood status	5 (20%)
Social well-being	1	Developing new relationships	13 (52%)
Self-regulation	1	Organising the time	5 (20%)
**The disadvantages of doing the PA**			
Physical harm	1	Increasing the risk of injury	16 (64%)
	2	Fears of PA addiction and obsession	5 (20%)
Social issues	1	Sometimes not attending family gatherings	5 (20%)

Also to raise the level of fitness while carrying heavy things, walking and climbing the stairs, so I do not feel tired. (P 19, aged 18–24, single, undergraduate)

The benefit of physical activities helped me to lose weight slightly, but in long periods, this type of exercise is a better way from losing weight rapidly to get a perfect and tight body. (P 22, aged 25–35, married, postgraduate)

In general, I find physical activity is a perfect way to reduce the risk of chronic diseases and a primary line of prevention against chronic diseases and to maintain health. (P 5, aged 18–24, single, undergraduate)

The second highest theme was psychological well-being. Four beliefs were endorsed: reducing stress and anxiety, increasing self-confidence, relieving negative feelings, and improving mood status. For instance, one interviewee stated the following:

Physical activities are a way to release my worries and stress such as study and work stress. (P 6, aged 18–24, single, undergraduate)

Within the social well-being theme, developing new relationships was endorsed by almost half of the sample. For the self-regulation theme, the interviewees mentioned that participating in PA made them more structured:

Physical activities help me in organizing my daily life and priorities. (P 9, aged 18–24, single, undergraduate)

Physical harm was the most frequently endorsed harmful consequence; the interviewees felt PA carries some risk of injury. Others disagreed; they thought the risk for injury is related only to the lack of knowledge and experience:

There are no disadvantages related to exercising, but the person may have exercise injuries. (P 17, aged 18–24, single, undergraduate)

I do not see any disadvantage related to exercising, but there might be if the person practice it without having enough knowledge that may hurt the person. (P 2, aged 25–35, single, undergraduate)

The fear of being addicted and obsessed with a rigid PA routine was considered as a typical threat to engage in an active lifestyle, with just under a fifth of the individuals raising this theme:

I do not see any disadvantages, but some people become obsessed about exercising too much, and this may hurt them in the end. (P 9, aged 25–35, single, undergraduate)

The social issues theme identified negative consequences by some participants. They claimed that skipping family gatherings influenced participation in PA:

The social lifestyle does not recognize the importance of women exercises that is why sometimes I do not attend family gatherings because I am busy with exercising or any physical activity that makes some of them upset with this kind of behaviour. (P 18, aged 25–35, single, postgraduate)

### Normative beliefs

Seven salient social referents were considered under four main themes, as shown in Table IV.

**Table IV.** Salient normative beliefs of young Saudi females aged 18–35 years regarding physical activity based on global guidelines.

Themes	Rank	Sub-themes	Frequency %
**Social referents perceived as approving or supporting PA**			
Family	1	Sister	12 (48%)
	2	Mother	10 (40%)
	3	Father	9 (36%)
	4	Brother	8 (32%)
Society	1	Friends	18 (72%)
Social media users	1	Social media influencers	15 (60%)
**Social referents perceived as disapproving or not supporting PA**			
Society	1	Saudi Community	6 (24%)

Individual family member opinions were a significant subjective belief endorsed by interviewees. Comments included the following:

My sisters play an important role in encouraging me to exercise. (P 6, aged 18–24, single, undergraduate)

My mother always encourages me to walk when the weather becomes good. (P 13, aged 18–24, single, undergraduate)

A third regarded their father and brother as significant influencers.

Society was the most endorsed theme, influencing either positively or negatively. Most of the participants acknowledged their friends as a major factor in their physically active decisions:

My friends have a role in motivating me to exercise. (P 24, aged 18–24, single, undergraduate)

Almost a quarter of respondents reported that society does not accept Saudi women’s involvement in PA, and others experienced no disapproval at all:

There are still some barriers in society in accepting the idea of women’s exercising, especially in the street. (P 10, aged 25–35, single, postgraduate)

I have not faced people who oppose or criticize my exercise. (P 1, aged 18–24, single, undergraduate)

Fifteen participants (60% endorsed) agreed that following and watching “leading influencers” on social media platforms engaging in a PA lifestyle or discussing the value of PA helped them feel that they also should engage:

Trainers, interested people in exercise and nutrition in social media, such as “Jumnaz;” she has an app targeting all categories of the society, whether people who suffer from obesity or people who want to gain weight. There are others such as Rahaf Fitness, Hamad AlRiseeni and Ninja Diet. (P 11, aged 18–24, single, undergraduate)

### Control beliefs

Thirteen salient control beliefs were considered under seven themes, as shown in Table V.

**Table V.** Salient control beliefs of young Saudi females aged 18–35 years regarding physical activity based on global guidelines.

Themes	Rank	Sub-themes	Frequency %
**Salient enablers beliefs to engage in PA**			
Natural environment enablers	1	Fairweather	15 (60%)
Built environment enablers	1	Availability of suitable parks	14 (56%)
	2	Having female gyms	5 (20%)
Personal enablers	1	Online sources	12 (48%)
	2	The ability to get affordable exercise equipment in the house	6 (24%)
**Salient barriers beliefs to engage in PA**			
Natural environment constraints	1	Hot weather	12 (48%)
Built environment constraints	1	Expensive gym membership	16 (64%)
	2	Lack of public transportation in cities	13 (52%)
	3	Lack of suitable walkways	11 (44%)
	4	Lack of enough female gyms	8 (32%)
Society constraints	1	Lack of social support	8 (32%)
Personal constraints	1	Lack of time	6 (24%)
	2	Wearing abaya while doing outdoor physical activity	5 (20%)

Within the environment enablers theme, fair weather, availability of appropriate parks, and female gyms were the most favoured natural and built enablers, respectively. Comments included the following:

When the weather is nice and fair outside, it motivates me to exercise. (P 25, aged 25–35, single, postgraduate)

I also prefer walking in parks when there is no one, so since the parks are open all day, it gives me the motivation to exercise. (P 5, aged 18–24, single, undergraduate)

Because of the existence and closeness of parks in the neighbourhood, currently, a lot of women’s gyms are available after the 2030 vision. (P 22, aged 25–35, married, postgraduate)

Numerous participants reported personal enablers. For example, almost half of the participants believed that the wide variety of workout materials on the Internet facilitated engagement in an active lifestyle, while almost a quarter of the participants viewed that having *a* home exercise room may afford convenience and enhance motivation. Comments included the following:

I find a lot of physical activities information in social media apps such as Snapchat, Instagram and Twitter. (P 9, aged 25–35, single, undergraduate).

Also the ability to own equipment at home with excellent prices and high quality. (P 6, aged 18–24, single, undergraduate)

For salient barriers beliefs, inappropriate infrastructure, costly facilities, and hot weather were the most identified barriers to support active living, respectively. Comments included the following:

The gyms are rare, far, and the prices of the subscriptions are high compared to men’s gym. (P 11, aged 18–24, single, undergraduate)

Public transportations are not activated yet, and this considered a barrier for me to exercise. (P 5, aged 18–24, single, undergraduate)

The roads are not well prepared for walking except for some main streets. (P 21 and P 7)

When the weather is hot, I lose enthusiasm for walking outside. (P 22, aged 25–35, married, postgraduate)

For society and personal constraints, almost half of the interviewees mentioned their PA level is likely to be influenced by social restrictions and traditions. Lack of time was more limiting for nearly a quarter of women, while others believed that wearing abaya is a barrier to participate in outdoor activities except walking. Comments included the following:

As well as cultural heritage, our society is conservative and masculine. It does not accept the idea of women exercising outside except walking. (P 22, aged 25–35, married, postgraduate)

Abaya is considered a barrier to me; even the new style of abayas made for sports is not suitable. (P 4, aged 18–24, single, undergraduate)

## Discussion

This study aimed to identify salient beliefs influencing young Saudi females’ participation in PA. Comprehending these beliefs is helpful because people have different thoughts and feelings about PA (Downs & Hausenblas, 2005). These beliefs can be used to promote an active lifestyle and change inactive behaviour in young Saudi females. There is little research on the specific beliefs that motivate young females to engage in PA at the suggested level.

This elicitation study shows that young Saudi women endorse 35 salient beliefs when forming attitudes, subjective norms, and perceived behavioural control. These beliefs are comprised of 17 themes that illustrate behavioural, normative, and control beliefs. These findings provide insight into potential salient personal as well as environmental barriers and enablers influencing young Saudi females’ engagement in PA behaviour with the adoption of a healthy lifestyle based on global WHO guidelines. The study identifies salient behavioural consequences, social referents, and circumstance beliefs among young Saudi women. This set of beliefs is discussed in more detail below.

### Salient behavioral beliefs

Examination of behavioural beliefs describes the cognitive underpinnings of young Saudi women’s attitudes towards an active lifestyle. Researching the underlying cognitive foundation enables an understanding of why individuals have such behavioural attitudes.

The need to improve body image and strength, increase self-confidence, enhance mood status, and organize time was identified as a positive salient consequence of PA that has not been unreported in Saudi women (Al-Harbi & Al-Harbi, 2017; Saaty et al., 2015).

This finding is consistent with other evidence that women’s concern about their physical appearance changes as observed in 10 Gulf region countries, including KSA, impacted by Western beauty ideals upon women and men (Calogero et al., 2007). In the Saudi context, female body weight and shape are a subject of daily discussion, uncommon in prior generations (Alqutub, 2018). Our findings indicate young Saudi women appear to engage in PA based upon a belief that it provides beauty benefits and that this new belief has likely arisen from Western influence.

PA has been described as the “best buy in public health” (Morris, 1994) due to the role of an active lifestyle in preventing the main causes of chronic disease and lowering direct and indirect health-related expenses. This qualitative study identifies young women as knowledgeable regarding the health benefits of PA; given the identification of two positive salient consequence beliefs of PA, lowering the risk of chronic diseases and enhancing health match those observed in a previous study of Saudi female adolescents (Janghorban et al., 2014). Psychological benefits related to stress reduction and negative feelings relief mirror the findings from the KSA (Al-Harbi & Al-Harbi, 2017) and the USA (Saaty et al., 2015).

On the other side, some participants thought that lack of knowledge and expertise is the root of workout-related injuries and that PA in itself does not result in injury. The fear of PA injuries might be because the PA education programme was introduced for the first time in Saudi female schools at the start of the school year 2017/2018 (Al-Hazzaa & AlMarzooqi, 2018). Accordingly, before 2017, young Saudi women’s healthy lifestyle practices might have emerged only during university years and among those who have university education (Majeed, 2015). This result has not been described previously (Al-Harbi & Al-Harbi, 2017). However, our finding is similar with the US study that found fear of getting hurt and lack of health insurance for injury were the most salient negative beliefs about PA engagement (Saaty et al., 2015). It seems possible that these beliefs about injuries are due to gender differences. Women are more prone to PA injuries than men (Murphy et al., 2003). Accordingly, safety concerns should be addressed when implementing PA intervention approaches.

Lack of time is a common hurdle for everyone, but there may be minor cultural differences at national and international levels. Interestingly, in the current study, many interviewees found that engaging in routine PA helps them to be organized. However, lack of time was a barrier for others within this community (Majeed, 2015). This combination of findings suggests health promoters would do well to promote the use of PA as a time management tool.

An unexpected discovery was the presence of fear of PA addiction. Typically, individuals develop positive PA addiction due to positive psychological effects (Lichtenstein et al., 2017). However, the interviewees feared engaging in overwhelming, life-threatening PA behaviour with negative effects. If working out becomes uncontrollable, it will be driven by fear, not pleasure. The volume and intensity of workouts is not an indicator of workout addiction; instead, harmful physical, psychological, and social consequences are the issue (Lichtenstein et al., 2017). More research into these perceptions is required for future PA promotion. Nonetheless, the current finding has important implications for developing exercise addiction treatment with prevention and counselling in exercise settings to play a role in addressing this issue at an early stage.

### Salient normative beliefs

This study describes the cognitive underpinnings of Saudi women’s subjective norms regarding PA through an examination of normative beliefs. Understanding the underlying cognitive foundation will allow a better understanding of how referents affect participants’ behaviour with respect to physical activity.

Numerous normative beliefs of young women found in this study appear similar to previously identified adolescents’ beliefs (Al-Harbi & Al-Harbi, 2017). These include the importance of the opinion of mothers, sisters, and friends. A possible explanation of maternal influence is that mothers in Muslim and especially Arab families are held in very high esteem; thus, their views and opinions play a very influential role in their family members’ lives. Importantly, female friends and sisters emerged as the consistent source of support associated with PA.

The current study confirms earlier findings that fathers and brothers are essential referents for the engagement in PA. In contrast to past findings (Aljehani et al., 2022), our results show that family members appear to play a limited role in fostering PA behaviour, but do not directly engage in it with young women. This might be due to the traditional, religious, and family-oriented nature of the Saudi society (Al-Hazzaa, 2018). Therefore, supportive family influence plays an essential role in encouraging PA behaviour in Saudi women but, beyond that, is limited. This finding contrasts with family approval and the social restrictions reported in previous studies as the leading barrier (80% of Saudi females with a mean age of 32.7 ± 9.8 years) to leisure time PA (Amin et al., 2011). Lack of social support and support from friends was also described as a barrier to being active among Saudi female (≥15 years old) (Al-Otaibi, 2013; AlQuaiz & Tayel, 2009; Awadalla et al., 2014). However, with a small sample size, caution must be applied, as the future research could investigate the associations between different types and the role of family influence and PA behaviour. Although interviewees perceived their community would likely disapprove of their PA engagement, actual disapproval of their activities was seldom reported (Aljehani et al., 2022).

Another important result was that social media influencers were identified as important referents. This result indicates more study is necessary to comprehend the function and potential of social media influencers to promote PA and a healthy lifestyle, also identified in adolescent females (Al-Harbi & Al-Harbi, 2017). As with social media, related research detected social media fitness influencers’ positive impact on PA (Durau & Diehl, 2018). Thus, social media may also strengthen the proximal social network’s role as influencers of PA related decisions. Consequently, throughout the implementation of PA promotional strategies, attention must be given to proximal social networks and media influencers.

### Salient control beliefs

Investigation of control beliefs represents the cognitive underpinnings of young Saudi women’s perceived behavioural control of an active lifestyle. Studying the cognitive foundations would assist in understanding the impact of enablers and barriers to PA in the study population.

Control beliefs are fundamental in determining effective interventions as they aid health promoters’ comprehension of the impact of enablers and barriers to the PA. Some control enablers beliefs identified in this study were similar to those reported previously, such as online PA sources (Al-Harbi & Al-Harbi, 2017). It seems then the Internet is seen as a crucial tool for educating and enabling Saudi females to live in a healthy lifestyle, including PA (Al-Harbi & Al-Harbi, 2017). This result would provide a further possible explanation of why social media influencers impact Saudi female perceptions towards an active lifestyle.

The interviewees thought female gyms would enable PA as it would guarantee discretion and modesty. Similarly, Saaty et al. (Saaty et al., 2015) found, even in the USA, females believed the lack of privacy from men in coed gyms is a deterrent (Al-Harbi & Al-Harbi, 2017). Access to female gyms appears to be a problem in Saudi Arabia as the government has only recently licenced female gyms, so the cost of the gym membership is high and gym availability low (Al-Harbi & Al-Harbi, 2017). Cost and low availability were identified as hindrances both in the current study and previously (Al-Harbi & Al-Harbi, 2017; Samara et al., 2015).

Interestingly, the interviewees were more likely to state barriers to PA than enablers. Unpleasant temperature, the primary natural environment factor in Saudi Arabia, was identified as a significant deterrent. Hot weather has consistently been considered a barrier to PA among Saudi women (AlQuaiz & Tayel, 2009; Amin et al., 2011; Khalaf et al., 2013). The presence of high outdoor summer temperatures resulting in reduced outdoor PA has been previously described in areas of the USA and Emirates (Chan & Ryan, 2009). Although the weather cannot be changed, researching how weather and seasonality affect PA beliefs and associated behaviours could help policymakers and health advocates customize policies to increase adaptation and coping. Taken together, these results suggest future research should investigate the impact of weather and seasonality upon PA behaviour to inform the design of new health-promoting interventions.

The interviewees identified beliefs of built environmental restrictions such as insufficient availability of public transportation and pathways. In accordance with the present findings, a systemic analysis of Arab regions conducted by Sharara et al. (2018) found that the insufficient public transit systems and lack of suitable paths deter walking and jogging activities (Sharara et al., 2018). These results suggest a need to support healthy transportation choices such as commuting or travel to public transport. Fortunately, built environmental constraints can be rapidly transformed. For instance, a substantial favourable association has been discovered between neighbourhood attributes like the presence of sidewalks to promote regular worldwide patterns of adults’ running and walking involvement (Hulteen et al., 2017) and overall PA in the USA (Council, 2005). The participants’ comments support the need to enhance the built environment through increased footpaths with shaded places and more green spaces. Improving the built environment could raise PA levels for Saudis of all genders. Quantification of the built environment’s contribution to beliefs and behaviour change requires further research.

The perceived lack of social support as a hindrance to PA engagement was an important conclusion previously unreported. This finding was not reported previously in an elicitation study among Saudi female adolescent (Al-Harbi & Al-Harbi, 2017). However, a lack of social support was claimed as a barrier to being active among Saudi female (≥15 years old) (Al-Otaibi, 2013; AlQuaiz & Tayel, 2009; Awadalla et al., 2014). We hypothesize this may be because the concept of “active and athletic” Saudi women is still new to Saudi society. Potentially these beliefs could be addressed through educational campaign detailing the significant advantages of PA for general health and well-being. Further research is required to understand how modifying social support could potentially enable women active lifestyles.

Among control beliefs, some perceived the abaya “traditional clothing covering the body” as an obstacle to outdoor workouts. This finding was supported by Al-Harbi and Al-Harbi (Al-Harbi & Al-Harbi, 2017) and by Sharara et al (Sharara et al., 2018). However, this belief highlights a tension between the Islamic religion as a potential enabler and an abaya as a barrier. Islamic faith instructs and encourages the followers to take care of their spiritual, emotional, and physical health needs with being physically active. However, the cultural background has a significant influence on lifestyle. As such, Saudi women are expected to wear an abaya, which covers their everyday clothing when out in public. Psychosocial determinants of PA behaviour should be studied in more depth to guide the development of culturally appropriate resources. The design of intervention programmes and activities that support women and truly fit the Saudi community could be of benefit to overcoming this barrier.

In brief, in our study, we found many behavioural, normative, and control beliefs that young Saudi women hold about PA based on international standards. Our findings are consistent in many aspects, with two earlier studies (Al-Harbi & Al-Harbi, 2017; Saaty et al., 2015). However, this study expanded the existing knowledge base by identifying other salient beliefs that cause women’s engagement with insufficient PA.

Future work might replicate research about beliefs at a broader level in more diverse settings including urban versus rural areas to enhance the generalizability of the findings and to enable the health promoters and policymakers to get a more complete understanding of young Saudi women’s perceptions towards their active lifestyle based on the setting.

Strengths of this work include using strong qualitative methods in the cross-cultural context, using a clear and thorough translation procedure, and appropriate study design to preserve anonymity in a culturally sensitive atmosphere. This work contributes to the growing body of qualitative research in cross-cultural studies; data are gathered in languages other than English using theoretical framework, which has been introduced into Western civilizations, and results are given in English. This work has contributed to the theoretical literature and thereby enriches elicitation studies.

Various techniques were used towards demonstrating credibility, transferability, dependability, and confirmability. The credibility was enhanced by using audio-recorded interviews and taking notes, seeking participants’ feedback on the completed transcripts, and having a plan detailing the materials and translation process. The dependability was improved through the transparent coding technique, with a skilled outside researcher in PA examining the procedures and findings through the “peer-debriefing” process (Yilmaz, 2013). The transferability was enhanced by a detailed description of the adopted methods to allow ease of replication (Creswell & Poth, 2018). Lastly, confirmability was improved using open-ended interviews and an interview framework to minimize any potential bias. Additionally, the researcher team had no involvement in selecting the participants; thereby, the researcher bias was reduced.

Other strength of this study was it is conducted in KSU, which attracts students from all across the country, in the capital city, which reflects the diversity of the Saudi population with different backgrounds (KSU, 2018). The salient beliefs identified may not be generalizable to the broader community of young Saudi women as it is not a representative sample. Nevertheless, this study identifies beliefs that can be measured in a cross-sectional survey. In particular, the findings of this study might only apply to those who live in urban, not rural, areas due to the public and domestic services differences. However, the Saudi population is one of the most uniform populations (Congress, 2006). The sample size is significant to a qualitative work (*N* = 25 participants), and interviews were in Arabic, the mother language. This paper, then, examined a broad range of young Saudi women beliefs about PA behaviour.

Despite these strengths, some limitations are acknowledged. First, the generalizability of these results is subject to certain limitations. The study design is qualitative, so it is not necessarily generalizable to other settings (Leung, 2015). However, it may be claimed that the outcome of generalization in qualitative study is knowledge that may describe, clarify, and inform researchers in a specific context (Myers, 2000; Polit & Beck, 2010). As generalization in qualitative research occurs mainly at the analysis and interpretation stage (Polit & Beck, 2010), strategies were adopted to support analytic generalization using appraisal criterion introduced by Tong, Sainsbury (Tong et al., 2007), COREQ, which emphasizes methodology, and the evaluative criterion of Lincoln and Guba (Lincoln & Guba, 1985), which stresses the rigour of interpretation and transferability of findings.

## Conclusion

According to the TPB, interventions may indeed be targeted at one or more determinants, attitudes, subjective norms, or perceived behavioural control to change behaviour by comprehending the underlying salient beliefs. Modifications within determinants should lead to improvements in behavioural intentions and provide sufficient control over behaviour, leading to the establishment of different intentions under proper conditions (Ajzen, 2006). The conclusions of this study have several significant implications for future practice.

The current research proposes initiatives and steps to change the intentions and behaviours regarding moderate to vigorous physical activities of young Saudi women. Several dominant beliefs are established to demonstrate that young Saudi women have a variety of favourable and few negative behavioural ideas regarding healthy active lifestyles. The attitude driven behaviour, which has been expressed by more than half of the participants includes increasing fitness level, having an ideal body weight, improving body image, reducing the risk of chronic diseases, and developing new relationships. Additionally, this study advances our comprehension of normative driven behaviour for this population that is influenced by sisters, mothers, peers, and social media bloggers. Finally, it contributes knowledge regarding the control driven behaviour of young Saudi females that is mainly motivated by fair weather, parks, and online resources.

These research findings can inform and guide innovative health advocates to create and implement successful Vision 2030 activities aimed at addressing one or more determinants. The in-depth understanding of young Saudi females’ beliefs can guide promoters to create a supportive environment with evidence-informed interventions that are well-grounded and cater for their physical health and well-being. These results could help develop culturally appropriate national programmes and policies for boosting participation in PA. More specifically, this research describes women’s attitudes as well as beliefs and highlights the types of programmes and environmental support women would utilize. Overall, each elicited belief can be used to promote an active lifestyle and alter the passive behaviour of young Saudi females. This study’s negative beliefs could be targeted with health-promoting strategies to change the Saudi female’s inactive behaviour effectively. Positive beliefs and enablers could be reinforced.

## Supplementary Material

Supplementary 1_clean.docx

Title page final .docx

## Data Availability

The datasets generated during and/or analysed during the current study are available from the corresponding author on reasonable request.

## References

[cit0001] Ajzen, I. Behavioral interventions based on the theory of planned behavior. Retrieved from https://people.umass.edu/aizen/pdf/tpb.intervention.pdf. (2006)

[cit0002] Ajzen, I. (2006b). Constructing a theory of planned behavior questionnaire in brief description of the theory of planned behavior, 1–14. Available from: https://pdfs.semanticscholar.org/0574/b20bd58130dd5a961f1a2db10fd1fcbae95d.pdf.

[cit0003] Alahmed, Z., & Lobelo, F. (2017). Physical activity promotion in Saudi Arabia: A critical role for clinicians and the health care system. *Journal of Epidemiology and Global Health*, 7(S1), S7. 10.1016/j.jegh.2017.10.005 29801594 PMC7386445

[cit0004] Alahmed, Z., & Lobelo, F. (2018). Physical activity promotion in Saudi Arabia: A critical role for clinicians and the health care system. *Journal of Epidemiology and Global Health*, 7 Suppl 1(Suppl 1), S7–S15 [Vision. National Transformation Program 2020]. 29801594 10.1016/j.jegh.2017.10.005 PMC7386445

[cit0005] Al-Harbi, B. F., & Al-Harbi, M. F. (2017). Eliciting salient beliefs about physical activity among female adolescent in Saudi Arabia: A qualitative study. *Public Health International*, 2(4), 116–123.

[cit0006] Al-Hazzaa, H. M. (2018). Physical inactivity in Saudi Arabia revisited: A systematic review of inactivity prevalence and perceived barriers to active living. *International Journal of Health Sciences*, 12(6), 50. PMC6257875 30534044

[cit0007] Al-Hazzaa, H. M., & AlMarzooqi, M. A. (2018). Descriptive analysis of physical activity Initiatives for health promotion in Saudi Arabia. *Frontiers in Public Health*, 6, 329. 10.3389/fpubh.2018.00329 30488032 PMC6246731

[cit0008] Aljehani, N., Razee, H., Ritchie, J., Valenzuela, T., Bunde Birouste, A., & Alkhaldi, G. (2022). Exploring female University students’ participation in physical activity in Saudi Arabia: A mixed-methods study. *Frontiers in Public Health*, 10. 10.3389/fpubh.2022.829296 PMC8971611 35372244

[cit0009] Al-Nozha, M. M., Al-Hazzaa, H. M., Arafah, M. R., Al-Khadra, A., Al-Mazrou, Y. Y., Al-Maatouq, M. A., Khan, N. B., Al-Marzouki, K., Al-Harthi, S. S., Abdullah, M., Al-Shahid, M. S. (2007). Prevalence of physical activity and inactivity among Saudis aged 30-70 years. A population-based cross-sectional study. *Saudi Medical Journal*, 28(4), 559–568. https://www.smj.org.sa/index.php/smj 17457478

[cit0010] Al-Otaibi, H. H. (2013). Measuring stages of change, perceived barriers and self-efficacy for physical activity in Saudi Arabia. *Asian Pacific Journal of Cancer Prevention: APJCP*, 14(2), 1009–1016. 10.7314/APJCP.2013.14.2.1009 23621177

[cit0011] AlQuaiz, A. M., & Tayel, S. A. (2009). Barriers to a healthy lifestyle among patients attending primary care clinics at a University Hospital in Riyadh. *Annals of Saudi Medicine*, 29(1), 30. 10.4103/0256-4947.51818 19139617 PMC2813614

[cit0012] Alqutub, K. R. A. (2018). *Ashamed bodies: The struggle into Changing body ideals*. University of Leicester.

[cit0013] Alsahli, M. S. Benefits and barriers to physical activity among Saudi female university students in the Kingdom of Saudi Arabia and the United States. Unpublished master’s dissertation. Middle Tennessee State University, 2016. Retrieved from https://search.proquest.com/docview/1865922032/fulltextPDF/752FCDAFBE89496APQ/1?accountid=13380.

[cit0014] Alshaikh, M. K., Filippidis, F. T., Baldove, J. P., Majeed, A., & Rawaf, S. (2016). Women in Saudi Arabia and the prevalence of cardiovascular risk factors: A systematic review. *Journal of Environmental and Public Health*, 2016, 1–15. 10.1155/2016/7479357 PMC5061969 27777590

[cit0015] Amin, T. T., Al Khoudair, A. S., Al Harbi, M. A., & Al Ali, A. R. (2012). Leisure time physical activity in Saudi Arabia: Prevalence, pattern and determining factors. *Asian Pacific Journal of Cancer Prevention*, 13(1), 351–360. 10.7314/apjcp.2012.13.1.351 22502700

[cit0016] Amin, T. T., Suleman, W., Ali, A., Gamal, A., & Wehedy, A. A. (2011). Pattern, prevalence, and perceived personal barriers toward physical activity among adult Saudis in Al-Hassa, KSA. *Journal of Physical Activity & Health*, 8(6), 775–784. 10.1123/jpah.8.6.775 21832292

[cit0017] Andersen, L. B., Mota, J., & DiPietro, L. (2016). Update on the global pandemic of physical inactivity. *The Lancet*, 388(10051), 1255–6. 10.1016/S0140-6736(16)30960-6 27475275

[cit0018] Awadalla, N., Aboelyazed, A., Hassanein, M., Khalil, S., Aftab, R., Gaballa, I., & Mahfouz, A. A. (2014). Assessment of physical inactivity and perceived barriers to physical activity among health college students, south-western Saudi Arabia. *Eastern Mediterranean Health Journal*, 20(10), 596–604. Available from: http://www.emro.who.int/emh-journal/eastern-mediterranean-health-journal/home.html 25356690

[cit0019] Brislin, R. W. (1986). Research instruments. In W. Lonner & J. Berry (Eds.), *Field methods in cross-cultural research* (pp. 137–164). Sage Publications.

[cit0020] Bull, F. C., & Bauman, A. E. (2011). Physical inactivity: The “Cinderella” risk factor for noncommunicable disease prevention. *Journal of Health Communication*, 16(2), 13–26. 10.1080/10810730.2011.601226 21916710

[cit0021] Burnard, P. (1991). A method of analysing interview transcripts in qualitative research. *Nurse Education Today*, 11(6), 461–6. 10.1016/0260-6917(91)90009-Y 1775125

[cit0022] Calogero, R. M., Boroughs, M., & Thompson, J. K. (2007). The impact of Western beauty ideals on the lives of women and men: A sociocultural perspective. In V.Swami & A.Furnham (Eds.), Body beautiful: Evolutionary and sociocultural perspectives(pp.259-298). New York: Palgrave Macmillan.)

[cit0023] Cha, E. S., Kim, K. H., & Erlen, J. A. (2007). Translation of scales in cross‐cultural research: Issues and techniques. *Journal of Advanced Nursing*, 58(4), 386–95. 10.1111/j.1365-2648.2007.04242.x 17442038

[cit0024] Chan, C. B., & Ryan, D. A. (2009). Assessing the effects of weather conditions on physical activity participation using objective measures. *International Journal of Environmental Research and Public Health*, 6(10), 2639–54. 10.3390/ijerph6102639 20054460 PMC2790098

[cit0025] Chen, H. Y., & Boore, J. R. (2010). Translation and back‐translation in qualitative nursing research: Methodological review. *Journal of Clinical Nursing*, 19(1–2), 234–239. 10.1111/j.1365-2702.2009.02896.x 19886874

[cit0026] Congress. Country profile: Saudi Arabia. 2006.

[cit0027] Council, N. R. (2005). Does the built environment influence physical activity? EXAMINING the EVIDENCE 2005 [Available from: http://onlinepubs.trb.org/onlinepubs/sr/sr282.pdf.

[cit0028] Creswell, J., & Poth, C. (2018). *Qualitative inquiry & research design : Choosing among five approaches* (4th ed.). SAGE.

[cit0029] Ding, D., Lawson, K. D., Kolbe-Alexander, T. L., Finkelstein, E. A., Katzmarzyk, P. T., Van Mechelen, W., & Pratt, M. (2016). The economic burden of physical inactivity: A global analysis of major non-communicable diseases. *The Lancet*, 388(10051), 1311–1324. 10.1016/S0140-6736(16)30383-X 27475266

[cit0030] Dishman, R. K. (2008). Gene–physical activity interactions in the etiology of obesity: Behavioral considerations. *Obesity*, 16(3), 60–65. 10.1038/oby.2008.520 19037216

[cit0031] Dishman, R. K., Heath, G. W., & Lee, I. M. (2013). *Physical activity epidemiology* (2nd ed.). Human Kinetics.

[cit0032] Downs, D. S., & Hausenblas, H. A. (2005). Elicitation studies and the theory of planned behavior: A systematic review of exercise beliefs. *Psychology of Sport and Exercise*, 6(1), 1–31. 10.1016/j.psychsport.2003.08.001

[cit0033] Durau, J., Diehl, S. The effects of social media fitness influencers on attitude and behavioral intentions. Paper presented at the 17th International Conference on Research in Advertising; Valencia, Spain. 2018.

[cit0034] Elo, S., Kääriäinen, M., Kanste, O., Pölkki, T., Utriainen, K., & Kyngäs, H. (2014). Qualitative content analysis: A focus on trustworthiness. *SAGE Open*, 4(1), 1–10. 10.1177/2158244014522633

[cit0035] Francis, J., Johnston, M., Eccles, M., Walker, A., Grimshaw, J. M., Foy, R., Kaner, E. F. S., Smith, L., & Bonetti, D. (2004). Constructing questionnaires based on the theory of planned behaviour: A manual for Health Services Researchers. *Quality of life and management of living resources*. Centre for Health Services Research. http://openaccess.city.ac.uk/id/eprint/1735

[cit0036] Guthold, R., Stevens, G. A., Riley, L. M., & Bull, F. C. (2018). Worldwide trends in insufficient physical activity from 2001 to 2016: A pooled analysis of 358 population-based surveys with 1· 9 million participants. *The Lancet Global Health*, 6(10), e1077–e1086. 10.1016/S2214-109X(18)30357-7 30193830

[cit0037] Hulteen, R. M., Smith, J. J., Morgan, P. J., Barnett, L. M., Hallal, P. C., Colyvas, K., & Lubans, D. R. (2017). Global participation in sport and leisure-time physical activities: A systematic review and meta-analysis. *Preventive Medicine*, 95, 14–25. 10.1016/j.ypmed.2016.11.027 27939265

[cit0038] Icek Ajzen, M. F. (1980). *Understanding attitudes and predicting social behaviour*. Prentice-Hall.

[cit0039] Janghorban, R., Roudsari, R. L., & Taghipour, A. (2014). Skype interviewing: The new generation of online synchronous interview in qualitative research. *International Journal of Qualitative Studies on Health and Well-Being*, 9(1), 24152–24154. 10.3402/qhw.v9.24152 24746247 PMC3991833

[cit0040] Khalaf, A., Ekblom, Ö., Kowalski, J., Berggren, V., Westergren, A., & Al-Hazzaa, H. (2013). Female university students’ physical activity levels and associated factors—a cross-sectional study in southwestern Saudi Arabia. *International Journal of Environmental Research and Public Health*, 10(8), 3502–3517. 10.3390/ijerph10083502 23939387 PMC3774451

[cit0041] KSU, K. S. U. (2018). About KSU. Retrieved from http://ksu.edu.sa/en/about-ksu

[cit0042] Leung, L. (2015). Validity, reliability, and generalizability in qualitative research. *Journal of Family Medicine and Primary Care*, 4(3), 324–7. 10.4103/2249-4863.161306 PMC4535087 26288766

[cit0043] Lichtenstein, M. B., Hinze, C. J., Emborg, B., Thomsen, F., & Hemmingsen, S. D. (2017). Compulsive exercise: Links, risks and challenges faced. *Psychology Research and Behavior Management*, 10, 85–95. 10.2147/PRBM.S113093 28435339 PMC5386595

[cit0044] Lincoln, Y. S., & Guba, E. G. (1985). *Naturalistic inquiry*. Sage Publications.

[cit0045] Lox, C. L., Ginis, K. A. M., & Petruzzello, S. J. (2017). *The psychology of exercise: Integrating theory and practice*. Taylor & Francis.

[cit0046] Mabry, R., Koohsari, M. J., Bull, F., & Owen, N. (2016). A systematic review of physical activity and sedentary behaviour research in the oil-producing countries of the Arabian Peninsula. *BMC Public Health*, 16(1), 1–22. 10.1186/s12889-016-3642-4 27655373 PMC5031342

[cit0047] Majeed, F. (2015). Association of BMI with diet and physical activity of female medical students at the University of Dammam, Kingdom of Saudi Arabia. *Journal of Taibah University Medical Sciences*, 10(2), 188–96. 10.1016/j.jtumed.2014.11.004

[cit0048] Morris, J. N. (1994). Exercise in the prevention of coronary heart disease: Today’s best buy in public health. *Medicine and Science in Sports and Exercise*, 26(7), 807–14. 10.1249/00005768-199407000-00001 7934752

[cit0049] Murphy, D., Connolly, D., & Beynnon, B. (2003). Risk factors for lower extremity injury: A review of the literature. *British Journal of Sports Medicine*, 37(1), 13–29. 10.1136/bjsm.37.1.13 12547739 PMC1724594

[cit0050] Myers, M. (2000). Qualitative research and the generalizability question: Standing firm with Proteus. *The Qualitative Report*, 4(3), 9. 10.46743/2160-3715/2000.2925

[cit0051] Nikander, P. (2008). Working with transcripts and translated data. *Qualitative Research in Psychology*, 5(3), 225–31. 10.1080/14780880802314346

[cit0052] Polit, D. F., & Beck, C. T. (2010). Generalization in quantitative and qualitative research: Myths and strategies. *International Journal of Nursing Studies*, 47(11), 1451–8. 10.1016/j.ijnurstu.2010.06.004 20598692

[cit0053] Prince, S. A., Gresty, K. M., Reed, J. L., Wright, E., Tremblay, M. S., & Reid, R. D. (2014). Individual, social and physical environmental correlates of sedentary behaviours in adults: A systematic review protocol. *Systematic Review*, 3(1), 1–8. 10.1186/2046-4053-3-120 PMC4207904 25336300

[cit0054] Regmi, K., Naidoo, J., & Pilkington, P. (2010). Understanding the processes of translation and transliteration in qualitative research. *International Journal of Qualitative Methods*, 9(1), 16–26. 10.1177/160940691000900103

[cit0055] Saaty, A. H., Reed, D. B., Zhang, W., & Boylan, M. (2015). Factors related to engaging in physical activity: A mixed methods study of female University students. *Open Journal of Preventive Medicine*, 5(10), 416–25. 10.4236/ojpm.2015.510046

[cit0056] Samara, A., Aro, A., Al-Rammah, T. Y., & Nistrup, A. R. (2015). Lack of facilities rather than sociocultural factors as the primary barrier to physical activity among female Saudi university students. *International Journal of Women’s Health*, 279–286. 10.2147/IJWH.S80680 PMC4358666 25834468

[cit0057] Sharara, E., Akik, C., Ghattas, H., & Obermeyer, C. M. (2018). Physical inactivity, gender and culture in Arab countries: A systematic literature asses. *BMC Public Health*, 18(1), 639. 10.1186/s12889-018-5472-z 29776343 PMC5960209

[cit0058] Tomasone, J. R., Martin Ginis, K. A., Estabrooks, P. A., & Domenicucci, L. (2014). ‘Changing minds’: Determining the effectiveness and key ingredients of an educational intervention to enhance healthcare professionals’ intentions to prescribe physical activity to patients with physical disabilities. *Implementation Science: IS*, 9, 30. 10.1186/1748-5908-9-30 24581329 PMC3945607

[cit0059] Tong, A., Sainsbury, P., & Craig, J. (2007). Consolidated criteria for reporting qualitative research (COREQ): A 32-item checklist for interviews and focus groups. *International Journal for Quality in Health Care*, 19(6), 349–57. 10.1093/intqhc/mzm042 17872937

[cit0060] Trost, S. G., Blair, S. N., & Khan, K. M. (2014). Physical inactivity remains the greatest public health problem of the 21st century: Evidence, improved methods and solutions using the ‘7 investments that work’as a framework. *British Journal of Sports Medicine*, 48(3), 169–170. 10.1136/bjsports-2013-093372 24415409

[cit0061] University, K. S. 2020. الملخص الإحصائي Available from: https://statinfo.ksu.edu.sa/ar/statistical_summary#.

[cit0062] Vision. National Transformation Program 2020 2016. Available from: http://vision2030.gov.sa/sites/default/files/NTP_En.pdf.

[cit0063] Warburton, D. E., Nicol, C. W., & Bredin, S. S. (2006). Health benefits of physical activity: The evidence. *Canadian Medical Association Journal*, 174(6), 801–809. 10.1503/cmaj.051351 16534088 PMC1402378

[cit0064] World Health Organisation. 2018. Physical Activity. Available from: https://www.who.int/news-room/fact-sheets/detail/physical-activity.

[cit0065] Yilmaz, K. (2013). Comparison of quantitative and qualitative research traditions: Epistemological, theoretical, and methodological differences. *European Journal of Education*, 48(2), 311–25. 10.1111/ejed.12014

