# Supplementary material for: Investigating the beliefs of Saudi females regarding physical activity: a qualitative exploration
Source: Int J Qual Stud Health Well-being. 2023 Dec 21;19(1):2296696. doi: 10.1080/17482631.2023.2296696 (PMC10763862; doi:10.1080/17482631.2023.2296696)
Supplement: Supplementary 1_clean.docx [file ZQHW_A_2296696_SM1669.docx]

# Supplementary one interview questions and protocol

Interview protocol is established to ensure the interviewer does not forget any key points that should be addressed during the interview. It serves to help remind the interviewer to relay essential information to the interviewee, such as the purpose of the interview, the process and length of the conversation, what will happen to the data obtained, and any confidentiality limitations. This guide serves as a checklist that includes a welcome, explanation, and thank you.

Purpose:

The primary goal of this qualitative interview is to understand the participants' views, attitudes, and responses to a particular phenomenon or experience ([Thomas, et al., 2005](#_ENREF_279)). open-ended questions will be used in the interview to allow the participants the opportunity to express their views fully without interruption; it is the best choice with regard to coding, analysing qualitative data ([Polgar & Thomas, 2013](#_ENREF_233)), and reducing the bias ([Gall, 2007](#_ENREF_135)).

Recording the interview

Many interviewers in qualitative studies use audio recording to aid in the preparation of the interview transcripts. The files can be played back on a personal computer with the proper programming to ensure the information is secure by utilising a digital audio recording device. Using a recorder is a helpful tool for overcoming the natural restrictions of human memory ([Polgar & Thomas, 2013](#_ENREF_233)).

Before starting the interview

- Ensure Skype is operational and that both the interviewer and interviewee are in private rooms.
- Thank the interviewee for taking the time to attend the interview.
- Ensure the interviewee feels comfortable, understands all the information in the Information Sheet, and answered all questions regarding the study. Then, they explain the purpose of the interview and the process and the length of the interview.
- Ask if the interviewee has any further questions before commencing the interview.
- Inform the interviewee the recording will begin.
- Ensure that the interviewee understands the meaning of vigorous and moderate-intensity physical activity.

At the beginning of the interview

A series of fundamental demographic questions will be asked at the beginning of the interview:

- Are you currently a student at KSU?
- What is your age?
- What is your marital status? Are you married or single?
- Do you study full- or part-time?
- How many years of university studies have you completed?
- Is your degree a graduate or undergraduate?

Interview guide

- The interviewer should ensure that the interviewee is familiar with Vision 2030 and up to date on new transformations occurring in Saudi Arabia due to the launch of Vision 2030. The following scenario will explain some of the goals of Vision 2030 related to the PA:

One of the Vision 2030 goals is “creating a vibrant society with fulfilling lives” by creating a supportive environment to promote a healthy lifestyle and social well-being. Following Vision 2030, Princess Reema Al-Saud was appointed as head of the women’s section at the General Sports Authority to oversee physical activity (PA) and sports participation amongst females. This has been considered an exceedingly important signal for potentially significant female access to and engagement in physically active behaviour by removing many restrictions on Saudi women, such as driving bans, permission to enter sports stadiums, and participation in Olympic championships.

- To fulfil the purpose of the study, the interviewer will be flexible and will introduce the following paragraph, which explains the behaviour of interest:

For this research, the definition of PA will follow the global recommendations by the World Health Organization (2011) regarding achieving the recommended level of PA, which has been defining meeting any of the following criteria:

- 150 minutes of moderate-intensity physical activity (at this level of exercise, a person’s heartbeat has raised significantly, and they will sweat / at this level of training, a person can say some words without stopping for breath)

**OR**

- 75 minutes of vigorous-intensity physical activity (at this level of exercise, a person’s breathing is challenging and fast, and their heartbeat has raised significantly/ at this level of training, a person is unable to say some words without stopping for breath)

**OR**

- an equivalent combination of both

You may be aware that PA can be done in different contexts. Those guidelines can be incorporated into any of the following contexts: leisure-time activities, or daily routine activities (e.g., occupational, household, active transportation)

- The interview builds on the assumption that interviewees need to have a space to express their inner thoughts ([Thomas, et al., 2005](#_ENREF_279)). Therefore, interviewees will be reassured that there are no correct or incorrect answers (merely say whatever comes to your mind first). Furthermore, to encourage the interviewees to speak freely, the interviewer will adopt neutral body language and use their skills to ease interviewees ([Polgar & Thomas, 2013](#_ENREF_233)). Thus, developed probes will be used to ensure that the interviewer will get detailed answers from the participants.

There are no correct or incorrect answers. Say whatever comes to your mind first.

| - Behavioural beliefs questions: | |
| --- | --- |
| Question 1 | - What do you believe are the pros of doing physical activity? |
| Question 2 | - What do you believe are the cons of doing physical activity? |
| Question 3 | - What else comes to mind when you think about doing physical activity? |
| Probe for: | - Positive changes after performing physical activity. - Adverse changes after completing physical activity. - Advantages of participating in physical activity include staying fit, meeting new friends, feeling active, weight control, fitness, strength, having fun, and blood circulation. - Disadvantages of participating in physical activity include feeling tired, the risk of injuries, and wasting time. - Changes in behaviour and mood after completing the physical activity include feeling relaxed, relieving stress, feeling tense, or lacking the confidence to participate in physical activity. - Changes in the way/ how you carry out the rest of your day if you perform the daily-recommended level of physical activity. |

| - Normative beliefs questions: | |
| --- | --- |
| Question 1 | - Which people would approve or support you doing physical activity? |
| Question 2 | - Which people would disapprove of you doing physical activity? |
| Question 3 | - Are there any other groups/ people who come to mind when you think about engaging in physical activity? |
| Probe for: | - Do you feel the reactions or the attitude of family members, i.e., father, mother, brothers, sisters, and friends, influence your participation in physical activity? - Do you feel the reactions of relatives and neighbours impact your participation in physical activity? - Do you consider the Athletes as role model regarding participating in physical activity? - Are the community's reactions toward involvement in physical activity encourage you to be physically active or discouraging you, i.e., knowing Saudi culture is very conservative and that relatives may consider girls’ outside exposure for such activities to be shameful. |

| - Control beliefs questions | |
| --- | --- |
| Question 1 | - What circumstances would enable you to be physically active? |
| Question 2 | - What circumstances would deter you from performing physical activity? |
| Question 3 | - Are there any other issues that come to mind when you think about the difficulty of engaging in physical activity? |
| Probe for: | - How easy or difficult is it for you to participate in physical activity?   What makes it easy or challenging to engage in physical activity? i.e., time, availability or lack of equipment, availability or lack of physical activity facilities, availability or lack of transportation, weather circumstances, injuries, and psychological circumstances. |

Closing the Interview

- Ask the interviewee if there is any further information they would like to add before ending the interview.
- Inform the interviewee that you are about to stop the recording.
- The interviewer asks the interviewee if they want to review the transcript of the interview for verification, either by sending the transcript to their Skype account or to their email, which they will need to provide.
- Thank them for taking part in the project.

Disconnect Skype, download and save the record as per data management procedures.
