# Supplementary material for: Investigating the beliefs of Saudi females regarding physical activity: a qualitative exploration
Source: Int J Qual Stud Health Well-being. 2023 Dec 21;19(1):2296696. doi: 10.1080/17482631.2023.2296696 (PMC10763862; doi:10.1080/17482631.2023.2296696)
Supplement: Title page final .docx [file ZQHW_A_2296696_SM1668.docx]

**Investigating the Beliefs of Saudi Females Regarding Physical Activity: A Qualitative Exploration**

***Authors***

Basmah Fehaid H Alharbi^1^, Philip Baker^2^, Toby Pavey^3^, Manal F. Alharbi^4^,

***Institutional addresses***

^1^ Qassim University, Applied Medical Science College, Basic Health Science Department, Al-Qassim Province, Kingdom of Saudi Arabia. The research field is in Epidemiology and Community Health, epidemiology, Public Health, Physical Activity, Microbiology and Evidence-based practice. <https://orcid.org/0000-0001-6863-469X>

^2^ Queensland University of Technology, Faculty of Health, School of Public Health and Social Work, Victoria Park Road, Kelvin Grove, QLD 4059. Extensive experience in Epidemiology in Public Health, University and Hospital settings. <https://orcid.org/0000-0002-2834-5436>

^3^ Queensland University of Technology, Faculty of Health, School of Exercise and Nutrition Sciences, Victoria Park Road, Kelvin Grove, QLD 4059. The research field is in Human Movement and Sports Science, Public Health, and Health Services. <https://orcid.org/0000-0002-4946-6683>

^4^ Maternal & Child Health Nursing Department, College of Nursing, King Saud University

Riyadh 11421, Saudi Arabia. The research field is in multidisciplinary and involves Mixed Methods, Qualitative Research, Physical Activity, Quality of Life and Cultural Issues. <https://orcid.org/0000-0001-6630-7476>

***Corresponding author:***

Manal F. Alharbi email: [Maalwahbi@ksu.edu.sa](mailto:Maalwahbi@ksu.edu.sa) Maternal & Child Health Nursing Department, College of Nursing, King Saud University-Riyadh, Saudi Arabia.

**Acknowledgements**

The researchers would like to thank the Deanship of Scientific Research, Qassim  University for funding the publication of this project. We would like to thank all study participants for sharing their perspectives with us.

**Authors' contributions**

**BA:** Study design, material preparation, ethics clearance, data collection, and write the original draft. **PB:** Contributed significantly to design research methods, instruments, ethics clearance, edit manuscript and supervision. **TP:** Contributed significantly to the design research methods, instruments, writing and review of this manuscript. **MA:** Ethics clearance (KSU), interviews conduction, scientific review and submit of this manuscript. All authors read and approved the final manuscript.

**Declarations**

**Availability of data and materials**

The datasets generated during and/or analyzed during the current study are available from the corresponding author on reasonable request.

**Funding**

This study was supported by a scholarship from Qassim University in Saudi Arabia as part of the PhD thesis. In addition, Queensland University of Technology provided financial assistance.

**Disclosure of interest**

The authors report no conflict of interest.

**Biographical note:**

Author 1: Assistant Professor, research field is Epidemiology and Community Health, Epidemiology, Public Health, Physical Activity, Microbiology and Evidence-based practice. <https://orcid.org/0000-0001-6863-469X> Author 2: Professor with extensive experience in Epidemiology in Public Health, University and Hospital settings. <https://orcid.org/0000-0002-2834-5436> Author 3: Associate professor. The research field is Human Movement and Sports Science, Public Health, and Health Services. <https://orcid.org/0000-0002-4946-6683> Author 4: Associate professor. The multidisciplinary research field involves Mixed Methods, Qualitative Research, Physical Activity, Quality of Life and Cultural Issues. <https://orcid.org/0000-0001-6630-7476>
